# Hospital Environment as a Source of Azole-Resistant *Aspergillus fumigatus* Strains with TR34/L98H and G448S Cyp51A Mutations

**DOI:** 10.3390/jof7010022

**Published:** 2021-01-02

**Authors:** Irene Gonzalez-Jimenez, Jose Lucio, Maria Dolores Menéndez-Fraga, Emilia Mellado, Teresa Peláez

**Affiliations:** 1 Mycology Reference Laboratory, National Centre for Microbiology, Instituto de Salud Carlos III (ISCIII), 28222 Majadahonda, Madrid, Spain; irene.gonzalez@isciii.es (I.G.-J.); jose.lucio@isciii.es (J.L.); 2 Hospital Universitario Central de Asturias, Fundación para la Investigación Biosanitaria del Principado de Asturias (FINBA), 33011 Oviedo, Asturias, Spain; mariadol.menendez@gmail.com (M.D.M.-F.); mtpelaez@gmail.com (T.P.); 3 Spanish Network for Research in Infectious Diseases (REIPI RD16/CIII/0004/0003), ISCIII, 28222 Majadahonda, Madrid, Spain

**Keywords:** *Aspergillus fumigatus*, azole resistance mechanisms, *cyp51A*, hospital environment

## Abstract

Azole-resistant *Aspergillus fumigatus* is an emerging worldwide problem with increasing reports of therapy failure cases produced by resistant isolates. A case of azole-resistant *A. fumigatus* hospital colonization in a patient is reported here. Investigations of the hospital environment led to the recovery of *A. fumigatus* strains harboring the TR34/L98H and the G448S Cyp51A azole resistance mechanisms. Isolate genotyping showed that one strain from the environment was isogenic with the patient strains. These are the first environmental *A. fumigatus* azole resistant strains collected in a hospital in Spain; it supports the idea of the hospital environment as a source of dissemination and colonization/infection by azole resistant *A. fumigatus* in patients. The isolation of an azole-resistant strain from an azole-naïve patient is an interesting finding, suggesting that an effective analysis of clinical and environmental sources must be done to detect azole resistance in *A. fumigatus*. The emergence and spread of these resistance mechanisms in *A. fumigatus* is of major concern because it confers high resistance to voriconazole and is associated with treatment failure in patients with invasive aspergillosis.

## 1. Introduction

Infections caused by the saprotrophic mold *Aspergillus fumigatus* and other *Aspergillus* spp. are due to the inhalation of conidia, which are present in the environment [1]. Daily, up to 200 conidia per person are inhaled, causing a wide spectrum of clinical affectations depending on the immunological status of the host [2]. In healthy immunocompetent individuals, the immune system is able to clean conidia from the lungs;however, immunosuppressed patients have a high predisposition to develop clinical manifestations associated with a worst outcome [1].

Clinical manifestations caused by *A. fumigatus* are encompassed under the name of aspergillosis, ranging from minor pathologies up to more severe forms, such as invasive pulmonary aspergillosis (IPA), with mortalityrates reaching 95% in immunocompromised hosts [3]. IPAand other forms of aspergillosis are currently being treated with a class of antifungal compounds named azoles [1,4]. Among azoles, the triazoles voriconazole, itraconazole, posaconazole, and isavuconazole are the most widely used drugs for both aspergillosis treatment andprophylaxis [5].

Lately, as is happening with a wide range of microorganisms worldwide, clinical isolates of *A. fumigatus* resistant to azoles are being acknowledged, and the prevalence is gradually increasing [6,7] involving a major concern due to its association with treatment failure in patients with IPA [8]. To date, *A. fumigatus* azole resistance is mostly associated with modifications of the azole target site, the enzyme Cyp51A encoded by the gene *cyp51A*, and its overexpression [9,10]. Triazole resistance can evolve during azole therapy in the clinical setting, but resistant isolates are also being detected in azole-naïve patients, suggesting an environmental origin of some resistance mechanisms [11].

So far, reports of modifications in *cyp51A,* or its expression, associated with azole resistance in *A. fumigatus* isolates can be distinguished in two categories. On the one hand, point mutations in the coding sequence of the gene involving amino acid changes in the protein (G54, P216, M220, G138, G448), and on the other hand tandem repeat insertions in the promoter region of the gene combined, or not, with point mutations in the coding sequence (TR34/L98H, TR53, TR34/R65K/L98H and TR46/Y121F/T289A) [10,12].Point mutations have usually been described in clinical isolates after long-term azole therapies while tandem repeat resistance mechanisms are more often isolated from environmental samples or azole-naïve patients [10].

Among all azole resistance mechanisms described to date, the most frequent is the TR34/L98H, associated with a multi-azole resistance phenotype to all clinical azoles. This mechanism was initially isolated in Europe, although it has spread worldwide since then [10,13,14]. Its isolation is normally linked to the use of azole fungicides to protect harvests by preventing crop damage [14] and, apart from the environment, strains harboring the TR34/L98H resistance mechanism have also been isolated from individuals, most of them azole-naïve patients that were originally infected by a strain carrying this mutation [13,15,16].

Cyp51A point mutations are frequently described in samples from patients that have undergone long-term azole treatments. G54 substitution is the most described in patients after treatment with itraconazole or posaconazole [17,18]. Other mutations in Cyp51Asuch asP216, M220, and G138P are occasionally described [9,10]. First isolated from a patient in 2003, the G448S mutationhas been the most frequently reported in patients under voriconazole treatment since 2009 [19,20,21,22,23,24,25,26,27,28,29]. In addition, strains bearing the G448S mutation have also been reportedfrom environmental sampling [30,31,32,33]. The susceptibility profile of *A. fumigatus* strains harboring this substitution shows resistance to voriconazole and isavuconazole and reduced susceptibility to itraconazole and posaconazole [19,20,21,22,23,34].

Here we report, for the first time, the isolation of environmental *A. fumigatus* azole resistantisolates in Spain. The azole resistance mechanismsof the isolates wereTR34/L98H and G448S inCyp51A. Moreover, the concomitant isolation of *A. fumigatus* azole resistant isogenic strains from a hospitalized patient and thehospital environment make the study more interesting.Whether the patient had a hospitalstrain acquisition or was the source of hospital contamination is discussed.

## 2. Materials and Methods

### 2.1. Aspergillus fumigatus Strains

Inthis study, a total offifteen *A. fumigatus* strains were analyzed, ten clinical and five environmental isolates.Strainsidentification was confirmed by amplification and sequencing of the ITS1-5.8S-ITS2 rDNA regions and a portion of β-tubulin gene [35].

### 2.2. Case Report and Environmental Search

In January 2019, a patient was admitted to the hospital with dyspnea, cough, and bronchial secretions. The patient had a background of hypertension, pneumoconiosis, and COPD. After ten days in the hospital, *A. fumigatus* was isolated in a sputum (15 January 2019) and no other pathogens were found in the sample. The patient had no obvious clinical signs of invasive aspergillosis, and this isolation was considered a colonization following the revised EORTC/MSG criteria [36]. Several colonies were analyzed (1003, 1003E, 1003E.2, 1004, 1004E, 1004E.2, 1005.1, 1005.2, 1005.3, and 1005.4). The calcofluor stain and lateral flow test were positive alerting the presence of *Aspergillus* species, and a quantitative real timePCR confirmed the identification of *A. fumigatus*. Two indoor environmental searches (23 January, 2019 and 5 February, 2019) of the patient hospital room and bathroom yielded *A. fumigatus*. On the first air sampling study 3 CFU/m^3^ fungal isolates were obtained and 4 CFU/m^3^ on the second. Five isolates in total were analyzed (TP1, TP2, TP3, TP4, and TP5). Volumetric air samples were obtained using a volumetric sampler (Merck Air Sampler MAS100) as previously described [37].

### 2.3. Cyp51AAmplification, PCR Conditions and Sequencing

For DNA extraction, conidia from each strain were cultured in glucose-yeast extract-peptone (GYEP) liquid medium (0.3% yeast extract, 1% peptone; Difco, Soria Melguizo, Madrid, Spain) with 2% glucose (Sigma-AldrichQuímica, Madrid, Spain) for 24 h at 37°C. After mechanical disruption of the mycelium by vortex-mixing with glass beads, genomic DNA of isolates was extracted using the phenol-chloroform method [38].

The full coding sequence of *cyp51A* including its promoter was amplified and sequenced. To exclude the possibility that any change identified in the sequences was due to PCR-induced errors, each isolate was independently analyzed twice. PCR reaction mixtures contained 0.5 μM of each primer, 0.2 μMofdeoxynucleoside triphosphate (Roche, Madrid, Spain), 5μLof PCR 10x buffer, 2 mMofMgCl_2_, DMSO 5.2%, 2.5 U of Taq DNA polymerase (Applied Biosystems, Foster City, CA, USA), and 100–200 ng of DNA in a final volume of 50μL. A DNA 1-kb molecular ladder (Promega, Madrid, Spain) was used for all electrophoresis analyses. Samples were amplified in a GeneAmp PCR System 9700 (Applied Biosystems, Foster City, CA, USA). The parameters used were 1 cycle of 5 min at 94°C and then 35 cycles of 30 s at 94°C, 45 s at 56°C for *cyp51A* promoter and 58 °C for *cyp51A* gene, and 2 min at 72 °C, followed by a 1 final cycle of 5 min at 72 °C. The amplified products were purified using IllustraExoProStar 1–step (GE Healthcare Life Science, Buckinghamshire, UK) and both strands were sequenced with the Big-Dye terminator cycle sequencing kit (Applied Biosystems, Foster City, CA, USA) following manufacturer’s instructions. All gene sequences were edited and assembled using Lasergene software package (DNAStar Inc., Madison, WI, USA). Primers used to amplify and sequence *cyp51A* and its promoter have been previously described [39].

### 2.4. Strains Genotyping

All of the strains included in this study were genotyped following the previously described typing method TRESPERG [40]. Four markers were used: (i) Afu2g05150 encoding an MP-2 antigenic galactomannan protein (MP2); (ii) Afu6g14090 encoding a hypothetical protein with a CFEM domain (CFEM); (iii) Afu3g08990 encoding a cell surface protein A (CSP) and (iv) Afu1g07140 (ERG), which encodes a putative C-24(28) sterol reductase. The combination of the genotypes obtained with each marker has a discriminatory value (D) of 0.9972 using the Simpson index.

### 2.5. Clinical Antifungal Drugs Susceptibility Testing

Antifungal susceptibility testing (AFST) was performed following the European Committee on Antimicrobial Susceptibility Testing (EUCAST) broth microdilution reference method 9.3.1 [41]. Antifungals used were amphotericin B (Sigma-Aldrich Química, Madrid, Spain) and the azoles itraconazole (Janssen Pharmaceutica, Madrid, Spain), voriconazole (Pfizer SA, Madrid, Spain), posaconazole (Schering-Plough Research Institute, Kenilworth, NJ, USA) and isavuconazole (BasileaPharmaceutica, Basel, Switzerland (tested from January 2017)). The final concentrations tested ranged from 0.03 to 16 mg/L for amphotericin B and 0.015 to 8 mg/L for the four azoles. *A. flavus* ATCC 204304 and *A. fumigatus* ATCC 204305 were used as quality control strains in all tests performed. Minimal inhibitory concentrations (MICs) were visually read after 24 and 48 h of incubation at 37 °C in a humid atmosphere. MICs were performed at least twice for each isolate. Clinical breakpoints for interpreting AFST results established by EUCAST [42] were used for classifying the *A. fumigatus* strains as susceptible or resistant.

## 3. Results

### 3.1. Amplification and Sequence Analysis of cyp51A

Amplification and sequencing of *cyp51A* including its promoter revealed two azole resistance mechanisms present in most (14/15) of the *A. fumigatus* strainsincluded in this study (Table 1). The first one consistingof a 34-bp tandem repeat insertion in the promoter region of *cyp51A* together with a L98H substitution in the coding sequence of the gene (TR34/L98H) that was present in all clinical samples and one environmental strain (TP3). The second one was a G448S substitution in *cyp51A,* which was harbored by three environmental samples (TP1, TP2, and TP4). Strain TP5 had no *cyp51A* promoter or the coding sequencemodifications (Table 1).

### 3.2. Strains Genotyping

Among all 15 *A. fumigatus* isolates included in this study, four genotypes were identified according to the TRESPERG typing assay (Table 1). Clinical isolates (1003, 1003E, 1003E.2, 1004, 1004E, and 1004E.2) and the environmental strain TP3 had the same genotype t10m1.1g08Ae05 named Type I. The four remaining clinical strains (1005.1, 1005.2, 1005.3, and 1005.4) had the genotype t02m1.1g09e16corresponding to Type II. Among the other four environmental strains collected, three of them (TP1, TP2, and TP4) were isogenic and characterized as Type III harboring the genotypet04Am1.3g05Ae07. Strain TP5 had a different genotype t04Am1.3g08Ae07 named Type IV. Strain CM2580 included as a wildtype susceptible strain had its own genotype different from the ones of the strains under study (Table 1).

### 3.3. Antifungal Susceptibility Testing

AFST to clinical azoles showed azole resistant MIC values in all strains tested except for the TP5 environmental strain which showed an azole-susceptible profile with ranges between 0.06 and 1 mg/L for all azoles tested. MIC values for amphotericin B were considered susceptible for all fifteen isolates. Two different azole resistance profiles were identified among all azole resistant isolates. All clinical strains and the TP3 environmental isolate had the same susceptibility profile consisting of>8 mg/L to itraconazole, 4 mg/L to voriconazole, 0.5 mg/L to posaconazole and 8 mg/L to isavuconazole. The other three environmental isolates (TP1, TP2, and TP4) had a MIC profile of 1 mg/L to itraconazole, 8 mg/mL to voriconazole, 0.25–0.5 mg/L to posaconazole and 4 mg/L to isavuconazole (Table 1).

## 4. Discussion

Azole-resistant *A. fumigatus* isolates from clinical and environmental origin are being acknowledged worldwide [10,14]. Resistant strainsharbordifferent resistance mechanisms that confer particular susceptibility profiles to antifungal drugs used in the clinical setting and also in the environment [16,43]. To date, the most prevalent azole resistance mechanism is the TR34/L98H combination in Cyp51A, the target for azole drugs, which has been mostly described from environmental isolates conferring multi-azole resistance [10]. In clinical samples the substitution G448S is frequently associated withvoriconazole and isavuconazoleresistance [19,20,21,22,23,34].

In this study, we isolated environmental azole-resistant samples of *A. fumigatus* for the first time in Spain. These strains were obtained from the environment of a hospital patient’s room, identifying two different resistance mechanisms (TR34/L98H and G448S) with two different genotypes. Out of the five samples obtained from the patient’s room four were azole-resistant and, out of these four, three harbored the resistance mechanism G448S and were isogenic.Theremaining strain had the resistance mechanism TR34/L98H and a different genotype. This confirms that two different azole-resistant *A. fumigatus* strains were isolated from the hospital room environment. The two azole resistance profiles found in this study are in agreement with studies previously performed for strains harboring the same Cyp51A resistance mechanisms [10,13,23].

Previous studies in Spain have analyzed *A. fumigatus* strains from clinical samples and, to date, only a few environmental samples have been studied [44,45]. Although no previous azole-resistant samples have been identified in the Spanish environment, environmental samples harboring the same TR34/L98H Cyp51A alteration have been previously collected in other geographic regions being the most common resistance mechanism found worldwide [15]. This resistance mechanism has been detected in many European countries (Germany, Denmark, France, The Netherlands, Italy, Ireland, UK, and Switzerland), Asia (China, India, Iran, Japan, North Korea, Thailand, and Taiwan), Africa (Tanzania), and America (Colombia, USA) [10].

The most remarkable result of this study is that all clinical strains obtained from the patient and the TP3 environmental sample obtained from the patient’s bathroom were isogenic, had the same MIC profile and Cyp51A resistance mechanism (TR34/L98H).Thissuggests that the patient had a hospital environmental acquisition of the strain, given that the houses and hospital environment can be contaminated by *A. fumigatus* azoleresistant strains [46,47]. Alternatively, the spread from patient to environment isa possibilityand this theory has been recently proposed by other authors [48,49]. A study performed in 2019 [49] was able to recover *A. fumigatus* from cough aerosolsof colonized patients with cystic fibrosis isogenic to those *A. fumigatus* obtained from the sputum of the same patient, suggesting environmental contamination through aerosols. Moreover, the case of a hospital patient acting as a source of *A. fumigatus* contamination of a hospital room environment after being infected in the same hospital, but different room has been reported recently [48].Other case reports from the beginning of the 2000s have described situations in which patients diagnosed with IPAhad isogenic strains with those isolated from the ICUs where they were hospitalized [50,51]. These studies bring to light the possibility of conidia being released through aerosols produced by aspergillosis patients, contaminating the air and causing patient-to-patient infection. Whether the patient of this studybecame colonized at the hospital by an *A. fumigatus* multi-azole resistant strain present in the environment, or if the patient was the source of an environmental contamination needs to be further investigated in order to elucidate the relation between these isogenic isolates.

Environmental strains harboring the point mutation G448S were also isolated in this study. This substitution is one of the most reported Cyp51A azole resistance mechanisms in patients treated with voriconazole (VCZ) [21,22,34,52,53,54].To date, the G448Smutation has been mainly reportedfrom the clinical setting all over the world including Europe, Japan, USA, and Australia [10,55].However, recent reports of environmental strains harboring this substitution are being acknowledged (Table 2), and a mutation believed to be unique to the clinical setting is now also observed in the environment in Spain and other geographical regions, suggesting a possible environmental origin which is starting to be proposed by some authors [56].

The selection of this G448S substitution in response to antifungal pressure has been reported in combination with a TR46/Y121F/M172I/T298A azole resistance mechanism [30,31] and also, the same substitution at the corresponding position has been detected in Cyp51As proteins from plant pathogen fungi that were exposed to azole drugs used in agriculture, conferring resistance to them [10]. All of these data suggest that the origin of the G448S mutation needs to be clarified since the clinical origin previously proposed is no longer so certain. In fact, the G448S mutations could have a dual selection, emerging under VCZ pressure in clinical settings or under triazole drugs used for crop protection.

## 5. Conclusions

In conclusion, our study demonstrates that azole resistant *A. fumigatus* are present in the hospital environment. Research on an *A. fumigatus* colonized patient room environment showed, for the first time in Spain, the isolation of *A. fumigatus* azole resistant strains, with two different genotypes and different resistance mechanisms (TR34/L98H and G448S). Strains bearing the azole resistance mechanism TR34/L98H, environmental or clinical, were isogenic. This interesting finding suggests that an effective analysis of environmental sources needs to be done in order to detect azole resistant *A. fumigatus*. Whether the patient had a hospital strain acquisition or was the source of hospital contamination is being investigated. If azole resistance spreads through cough aerosols from patient to patient, the spread from patient to environment is also a possibility. In addition, the isolation of environmental strains harboring the G448S resistance mechanism questions the origin of this mutation, it might emerge under either clinical or environmental selective pressure.

## Figures and Tables

**Table 1:** Minimal inhibitory concentrations (MICs), azole resistance mechanisms and TRESPERG typing assay of fifteen *A. fumigatus* strains. (AmB: amphotericin B; ITC: itraconazole; VCZ: voriconazole; POS: posaconazole; ISV: isavuconazole).

Strains	Source	Cyp51A Mutation	MICs (mg/L)					TRESPERG	Type
			AmB	ITC	VCZ	POS	ISV		
**1003**	Patient	TR34/L98H	0.5	>8	4	0.5	8	t10m1.1g08Ae05	I
**1003E**	Patient	TR34/L98H	0.5	>8	4	0.5	8	t10m1.1g08Ae05	I
**1003E2**	Patient	TR34/L98H	0.5	>8	4	0.5	8	t10m1.1g08Ae05	I
**1004**	Patient	TR34/L98H	0.5	>8	4	0.5	8	t10m1.1g08Ae05	I
**1004E**	Patient	TR34/L98H	0.5	>8	4	0.5	8	t10m1.1g08Ae05	I
**1004E2**	Patient	TR34/L98H	0.5	>8	4	0.5	8	t10m1.1g08Ae05	I
**1005.1**	Patient	TR34/L98H	0.125	>8	4	0.5	8	t02m1.1g09e16	II
**1005.2**	Patient	TR34/L98H	0.125	>8	4	0.5	8	t02m1.1g09e16	II
**1005.3**	Patient	TR34/L98H	0.125	>8	4	0.5	8	t02m1.1g09e16	II
**1005.4**	Patient	TR34/L98H	0.125	>8	4	0.5	8	t02m1.1g09e16	II
**TP1**	Bathroom	G448S	0.25	1	8	0.5	4	t04Am1.3g05Ae07	III
**TP2**	Room	G448S	0.5	1	8	0.25	4	t04Am1.3g05Ae07	III
**TP3**	Bathroom	TR34/L98H	0.5	>8	4	0.5	8	t10m1.1g08Ae05	I
**TP4**	Room	G448S	0.5	1	8	0.25	4	t04Am1.3g05Ae07	III
**TP5**	Room	WT	0.25–0.5	0.25	0.5–1	0.06	0.5–1	t04Am1.3g08Ae07	IV
**CM2580**	Control	WT	0.25–1	0.12–1	0.25–1	0.03–0.25	0.25–1	t01m5.5g03e11	--

**Table 2:** Reported *Aspergillus fumigatus* isolates harboring the mutation G448S in Cyp51A.

IsolationYear	Country	Origin	Cyp51A Mutation	N Isolates	Reference
2003	USA	Clinical	G448S	1	[19]
2003	USA	Laboratorymutants	G448S	5	[20]
2005	France	Clinical	G448S	1	[21]
2009	UK	Clinical	G448S	2	[22]
2011	Spain	Clinical	G448S	1	[23]
2012	USA	Laboratory mutants	G448S	6	[34]
2013	Australia	Clinical	G448S	1	[24]
2011–2015	USA	Clinical	G448S	4	[26]
2015–2016	Belgium	Clinical	G448S	1	[29]
2017	The Netherlands	Environmental	TR46/Y121F/M172I/T289A/G448S	4	[30]
2017	China	Laboratorymutants	N248K, G448S	1	[57]
2012–2019	Japan	Clinical	G448S	5	[25,27,28]
2015	The Netherlands	Environmental	TR92/Y121F/M172I/T289A/G448S	2	[31]
2020	Japan (The Netherlands)	Flowerbulbs	TR46/Y121F/M172I/T289A/G448S	7	[32]
2016	China	Environmental	G448S	9	[33]

## Data Availability

All sequence data and protocols associated with the publication are available to readers on request.
